# Risk factors of preterm birth among mothers who gave birth in public hospitals of central zone, Tigray, Ethiopia: unmatched case–control study 2017/2018

**DOI:** 10.1186/s13104-018-3693-y

**Published:** 2018-08-13

**Authors:** Girmay Teklay, Tsega Teshale, Hagos Tasew, Teklewoini Mariye, Hagos Berihu, Teklay Zeru

**Affiliations:** 1 School of Nursing, College of Health Science and Comprehensive Specialized Hospital, Aksum University, Po Box:-298, Axum, Tigray Ethiopia; 2 Department of Medical Laboratory, College of Health Science and Comprehensive Specialized Hospital, Aksum University, Axum, Tigray Ethiopia

**Keywords:** Preterm birth, Risk factors, Mothers, Central zone

## Abstract

**Objective:**

The aim of the study was to identify the risk factors for preterm birth in public hospitals of the central zone, Tigray, Ethiopia 2017/2018.

**Result:**

A total of 88 neonates who born preterm (cases) and 176 neonates who born term (controls) with their index mothers were included making a response rate of 100%. About 84/88 (95.5%) mothers in cases and 173/176 (98.3%) in control had antenatal care follow up. Among them, 33 (39.3%) cases and 102 (58%) controls were had antenatal care follow up four times and above. In multiple logistic regression at P-value < 0.05, mothers with ANC follow up less than four [AOR 95% CI 2.15 (1.19, 3.85)], mothers with pregnancy-induced hypertension [AOR 3.245; 95% CI (1.58, 6.67)], multiple pregnancy [AOR 2.47; 95% CI (1.14, 5.33)], fetal distress [AOR 4.0; 95% CI (1.9, 8.2)] and birth defect [AOR 3.19; 95% CI (1.22, 8.34)] were independent risk factors of preterm delivery.

## Introduction

Preterm birth is demarcated as a delivery which occurs at less than 37 completed weeks of gestation or fewer than 259 days [1]. It is classified as extremely preterm (< 28 weeks), very preterm (28 to < 32 weeks), and moderate (32 to < 34 weeks) and late preterm (34 to < 37 weeks) [2]. Preterm birth is a syndrome which can be classified into spontaneous preterm birth and provider-initiated preterm birth [2, 3].

Worldwide approximately 15 million babies are born too soon every year as a preterm birth and more than 60% of them were in sub-Saharan Africa and South Asia where health systems are weak in access and minimum health services utilization [4]. Even though there are many different causes of preterm-related neonatal death, greater than 75% of deaths are preventable without the need for intensive care [5, 6].

Preterm birth is the biggest cause of neonatal mortality worldwide and the second leading cause of underage five child deaths following pneumonia [4, 7, 8]. The incidence rates of preterm delivery were highest in Africa and North America, 11.9% and 10.6%, respectively [9].

There is an intense survival gap for premature babies depending on where they are born [3, 10]. Preterm birth is becoming the cause for economic instability in the family, community and the nation at large. It is not only resulted in economic burdens due to initial neonatal treatment but also in substantial costs to health services after discharge from the neonatal unit [3].

Being born preterm is a risk factor that put infants at higher risks of chronic diseases and death later in life [11]. Babies born preterm have continued to remain below the standard growth curve and demonstrate the reduced ability for catch-up growth in their life [12]. Moreover, preterm delivery and its consequences contribute to subsequent disabilities and neurological abnormalities to the infant’s potential that may persist all over their lives amongst survivors [2, 12, 13].

Socio-demographic factors, obstetric and gynecological-related factors, maternal medical disorder and common medical problems in neonates were some of them [9, 14–16]. Additionally, high levels of preterm birth in Africa are also possibly presented due to intrauterine infection or lack of availability of drugs, like Tocolytic agents [1].

Global progress for child survival and health cannot be achieved without addressing preterm birth [15]. This paper is important for policymaker by giving important information related to preterm birth to give intervention for prevention.

Despite the increase in ANC coverage and recommended guidelines for maternal and neonatal care, the number of preterm birth is increased through time. Even if there are limited studies which show the risk factors of preterm birth through the card review in some part of Ethiopia, the risk factors are different in different settings because of the difference in the healthcare system and demographic features. Consequently, this study was aimed to identify the risk factors for preterm birth in the central zone of Tigray.

## Main text

### Study area and period

The study was carried out in public hospitals of central zone Tigray, which has three hospitals named as St. Marry Hospital, Adwa Hospital, and Abyi Adi Hospital. We include 25 cases and 50 controls in Abyi Adi Hospital, 30 cases and 60 controls in Adwa Hospital and 33 cases and 66 controls in St. Marry Hospital. Data collection period was undertaken from December 2017 to February 2018.

### Study design

A hospital-based un-matched retrospective case–control study design was employed.

### Source population

All mothers who gave birth in public hospitals of the central zone of Tigray.

### Study population

*For cases* All mothers with alive PTB (28 to < 37 weeks) in public hospitals of the central zone of Tigray.

*For controls* All mothers with full-term birth (≥ 37 weeks) at the same hospitals.

### Sample size and sampling technique

A double population proportion formula (stat calc EPI info 7.1.1) was used with 95% confidence level, 80% power and proportion of controls with exposure of previous history of preterm birth 36.36% [17], odds ratio 2.25 and case to control ratio were 1:2. The final sample size with adjustment for non-response rate of 10% was 264 (88 cases and 176 controls). Systematic sampling technique was used every two intervals in both cases and controls. The first participant was selected by lottery method. Confirmation of preterm birth admission was taken from the documentation and information from the staffs.

### Data collection tools and procedures

Data was collected using interviewer-administered pretested semi-structured questionnaires and data recorded. The questionnaires were taken from different related studies [3, 16–18] and necessary modifications were done to be applicable to the current study and population. A translated Tigrigna version questioner was used to collect data. Four B.Sc. Nurses for data collection and two MSc nurses for supervisor were recruited.

### Data quality control

Training was given for data collectors and the questionnaires were pretested on 5% of the sample size at Suhul Hospital prior to the data collection period. Continuous follow-up and supervision were made by the supervisor and principal investigator. All participants were interviewed privately.

### Study variables

#### Dependent variable

- Preterm birth.

#### Independent variables

- Maternal socio-demographic factors, gynecologic and obstetric related factors, maternal medical history of disorders, maternal emotional stress, infant’s characteristics. Those information was obtained by face to face interview and record files.

### Operational definitions

*Preterm birth* Those who born in < 37 weeks.

### Diagnostic criteria for clinical conditions [19]

- *Gestational diabetes mellitus* Fasting plasma glucose ≥ 126 mg/dl.
- *Pregnancy-induced hypertension* Systolic blood pressure ≥ 130 mm Hg and/or Diastolic blood pressure ≥ 90 mm Hg.
- *Anemia* Hemoglobin < 11.0 g/dl.
- *Oligohydramnios* Amniotic fluid index < 8.

### Plan for data processing and analysis

The data was cleaned, entered and edited using EPIDATA version 3.1 statistical software, then exported to SPSS Version 22.0 for analysis. Results were presented using tables and texts. Hosmer–Lemeshow test was used to check the appropriateness of the model for analysis. Variables which were show statistical significance during bivariate analysis at P-value ≤ 0.25 were entered to multivariable logistic regression. Adjusted odds ratios (AOR) with 95% CI, were estimated to assess the strength of associations and statistical significance was declared at a P-value < 0.05.

## Result

### Socio-demographic characteristics of respondents

In this study, all the study participants were accepted to be part of the study which includes 88 preterm neonates (cases) and 176 term neonates (controls). The mean (± standard deviation) age of mothers was 27 ± 6 years’ ranges from 17 to 45 years. Most of the respondents 80 (90.9%) cases and 166 (94.3%) controls were orthodox. Regarding marital status, 79 (89.8%) cases and 161 (91.5%) of controls were married. About 60 (68.2%) of cases and 131 (74.4%) of controls were housewives by occupation and 25 (28.4%) cases and 46 (26.1%) of controls were not able to read and write (Table 1).

**Table 1:** Distribution of socio demographic characteristics among mothers who gave birth in public hospitals of central zone, Tigray, Ethiopia, 2018

Variable	Category	Cases	Control	Total
Residence	Urban	46 (52.3%)	98 (55.7%)	144 (54.5%)
	Rural	42 (47.7%)	78 (44.3%)	120 (45.5%)
Age of the mother	≥ 19	9 (10.2%)	16 (9.1%)	25 (9.5%)
	20–24	31 (35.2%)	51 (29%)	82 (31.1%)
	25–29	14 (15.9%)	53 (30.1%)	67 (25.4%)
	30–34	16 (18.2%)	31 (17.6%)	47 (17.8%)
	≥ 35	18 (20.5%)	25 (14.2%)	43 (16.3%)
Educational status	Can’t read and right	25 (28.4%)	46 (26.1%)	71 (26.9%)
	Read and right	8 (9.1%)	20 (11.4%)	28 (10.6%)
	Primary school	21 (23.9%)	39 (22.2%)	60 (22.7%)
	Secondary school	26 (29.5%)	46 (26.1%)	72 (27.3%)
	College and above	8 (9.1%)	25 (14.2%)	33 (12.5%)
Marital status	Unmarried	6 (6.8%)	10 (5.7%)	16 (6.1%)
	Married	79 (89.8%)	161 (91.5%)	240 (90.9%)
	Widowed/divorced	3 (3.4%)	5 (2.8%)	8 (3%)
Religion	Orthodox	80 (90.9%)	166 (94.3%)	246 (93.2%)
	Muslim	8 (9.1%)	10 (5.7%)	18 (6.8%)
Occupational status	Housewife	60 (68.2%)	131 (74.4%)	191 (72.3%)
	Governmental employee	10 (11.4%)	17 (9.7%)	27 (10.2%)
	Private employee	9 (10.2%)	10 (5.7%)	19 (7.2%)
	Daily/farmer/other	9 (10.2%)	18 (10.2%)	27 (10.2%)

### Maternal obstetrics and fetal condition

In our study 84 (95.5%) cases and 173 (98.3%) were had ANC follow up. Among them, 33 (39.3%) cases and 102 (58%) controls were had ANC follow up four times and above. Moreover, 64 (72.7%) newborn from the cases and 139 (79%) controls were born through Spontaneous vaginal delivery. About 50 (56.8%) cases and 50 (28.4%) of controls had one or more of pregnancy-related complications. Regarding the maternal emotional stress about 3 (3.4%) cases and 9 (5.1%) controls had maternal emotional stress, and 35 (39.8%) mothers in cases and 59 (33.5%) mothers in controls had lack of social support. About 13 (14.8%) cases and 19 (10.8%) of controls had one or more maternal medical problems. About 30 (34%) cases and 17 (9.7%) controls had fetal distress. All preterm neonates and 13 (12.9%) of term neonates had a birth weight of < 2.5 kg. Regarding the fetal presentation, 72 (81.8%) cases and 151 (85.8%) controls were in vertex presentation. From the total study participant, about 15 (17%) cases and 9 (5.1%) of controls had a birth defect (Table 2).

**Table 2:** Maternal obstetrics and fetal condition among mothers who gave birth in public hospitals of central zone, Tigray, Ethiopia, 2018

Variable	Category	Cases	Control	Total
ANC follow	Yes	84 (95.5%)	173 (98.3%)	257 (93.5%)
	No	4 (4.5%)	3 (1.7%)	7 (2.7%)
How many ANC follow	< 4	51 (60.7%)	74 (42%)	127 (48.1%)
	≥ 4	33 (39.3%)	102 (58%)	137 (51.9%)
Birth interval	No previous birth	42 (47.7%)	77 (43.8%)	119 (45.1%)
	12–24 months	3 (3.4%)	5 (2.8%)	8 (3%)
	24–36 months	15 (17.1%)	33 (18.8%)	48 (18.2%)
	≥ 36 months	28 (31.8%)	61 (34.6%)	89 (33.7%)
Parity	Prim gravida	41 (46.6%)	76 (43.2%)	117 (44.3%)
	Multigravida	47 (53.4%)	100 (56.8%)	147 (55.7%)
Immediate last mode of delivery	Spontaneous normal	40 (85.1%)	81 (81%)	121 (83.4%)
	Indicated	7 (14.9%)	19 (19%)	24 (16.6%)
Immediate previous pregnancy outcome	Preterm	7 (14.9%)	12 (12%)	17 (11.7%)
	Still birth	9 (19.1%)	10 (10%)	19 (13.1%)
	Normal birth	31 (66%)	78 (78%)	109 (75.2%)
Maternal RH factor	Positive	79 (89.8%)	152 (86.4%)	231 (87.5%)
	Negative	9 (10.2%)	24 (13.6%)	33 (12.5%)
Current mode of delivery	Spontaneous normal	64 (72.7%)	139 (79%)	203 (76.9%)
	Iatrogenic delivery	24 (27.4%)	37 (21%)	61 (23.1)
Current pregnancy has complication	Yes	50 (56.8%)	50 (28.4%)	100 (37.9%)
	No	38 (43.2%)	126 (71.6%)	164 (62.1%)
Type of current pregnancy complication (multiple options were possible)	Hypertension	30 (60%)	18 (36%)	48 (48%)
	Multiple pregnancy	22 (44%)	17 (34%)	39 (39%)
	Poly/oligo hydramnios	5 (10%)	9 (18%)	14 (14%)
	PROM	16 (32%)	15 (30%)	31 (31%)
	Meconium stained	6 (12%)	8 (16%)	14 (14%)
	Maternal fever	6 (12%)	10 (20%)	16 (16%)
	Cephalo-pelvic disproportion	1 (2%)	8 (16%)	9 (9%)
	Placental previa	1 (2%)	13 (26%)	14 (14%)
Maternal emotional stress	Yes	3 (3.4%)	9 (5.1%)	12 (4.5%)
	No	85 (96.6%)	167 (94.9%)	252 (95.5%)
Maternal social support	Lack of social support	35 (39.8%)	59 (33.5%)	94 (35.6%)
	Have social support	53 (60.2%)	117 (66.5%)	170 (64.4%)
Maternal medical problem	Yes	13 (14.8%)	19 (10.8%)	32 (12.1%)
	No	75 (85.2%)	157 (89.2%)	232 (87.9%)
Type of medical problem (multiple options were possible)	Anemia	11 (84.6%)	11 (57.9%)	22 (68.8%)
	Malaria	5 (38.5%)	10 (52.6%)	15 (46.9%)
	UTI	10 (76.9%)	13 (68.4%)	23 (71.9%)
Maternal height	≤ 150	7 (8%)	31 (17.6%)	38 (14.4%)
	> 150	81 (92%)	145 (82.4%)	226 (85.6%)
Maternal MUAC	< 19	20 (22.7%)	48 (27.3%)	68 (25.8%)
	≥ 19	68 (77.2%)	128 (72.7%)	196 (74.2%)
Sex infant	Male	53 (60.2%)	100 (56.8%)	153 (58%)
	Female	35 (39.8%)	76 (43.2%)	111 (42%)
Birth weight of infant	< 2500 g	88 (100%)	13 (7.4%)	101 (38.3%)
	≥ 2500 g		163 (92.6%)	163 (61.7%)
Fetal distress	Yes	30 (34%)	17 (9.7%)	47 (17.8%)
	No	58 (66%)	159 (90.3%)	217 (82.2%)
Fetal presentation	Vertex	72 (81.8%)	151 (85.8%)	223 (84.5%)
	Non vertex	16 (18.2%)	25 (14.2%)	41 (15.5%)
Congenital/birth defect	Yes	15 (17%)	9 (5.1%)	24 (9.1%)
	No	73 (83%)	167 (94.9%)	240 (90.9%)

### Risk factors associated with preterm birth

In the bivariate logistic regression at 25% level of significance, number of ANC follow up, maternal height, birth defect, fetal distress, PROM, multiple pregnancies, pregnancy-induced hypertension were statically significant with preterm birth.

In multiple logistic regression at P-value < 0.05 using backward elimination method, mothers with ANC follow up less than four were two times more to give preterm birth than those who had greater than or equal to four ANC follow up (AOR 95% CI 2.15 (1.19, 3.85). Mothers with pregnancy-induced hypertension were about three times higher to give preterm birth than those who had no hypertension [AOR 3.245; 95% CI (1.58, 6.67)]. The odds of a mother with multiple pregnancies were about two times more to give preterm birth than mothers with single pregnancy [AOR 2.47; 95% CI (1.14, 5.33)]. Neonate with fetal distress during labor and delivery were about four times higher to be preterm than those who had no fetal distress [AOR 4.0; 95% CI (1.9, 8.2)] and those who have birth defect were about three times higher to had a chance of preterm birth [AOR 3.19; 95% CI (1.22, 8.34)] (Table 3).

**Table 3:** Factors associated with preterm birth among mothers who gave birth in public hospitals of central zone, Tigray, Ethiopia, 2018

Variables	Category	Cases	Controls	COR (95% CI)	AOR (95% CI)
Number of ANC follow up	< 4	51 (60.7%)	74 (42%)	1.09 (1.24, 3.52)	2.15 (1.19, 3.85)**
	≥ 4	33 (39.3%)	102 (58%)	1	1
Pregnancy induced hypertension	Yes	30 (60%)	18 (36%)	4.54 (2.35, 8.76)	3.24 (1.58, 6.67)**
	No	20 (40%)	32 (64%)	1	1
Multiple pregnancy	Yes	22 (44%)	17 (34%)	3.12 (1.56, 6.25)	2.5 (1.14, 5.33)**
	No	28 (56%)	33 (66%)	1	1
Premature rapture of membrane	Yes	16 (32%)	15 (30%)	2.38 (1.12, 5.08)	1.02 (0.39, 2.68)
	No	34 (68%)	35 (70%)	1	
Maternal height	≤ 150 cm	7 (8%)	31 (17.6%)	0.4 (0.17, 0.96)	0.42 (0.16, 1.0)
	> 150 cm	81 (92%)	145 (82.4%)	1	1
Birth defect	Yes	15 (17%)	9 (5.1%)	3.81 (1.59, 9.11)	3.20 (1.22, 8.32)**
	No	73 (83%)	167 (94.9%)	1	1
Fetal distress	Yes	30 (34%)	17 (9.7%)	4.84 (2.48, 9.42)	4.0 (1.94, 8.23)**
	No	58 (66%)	159 (90.3%)	1	1

** Variable are significantly associated with preterm birth at P-value of < 0.05

### Discussion

The study was aimed to assess the risk factors for preterm birth in order to tackle the burden of the disease and its associated problems.

The lack of ANC diminishes the chance of identifying risks and provide appropriate interventions towards prevention of preterm delivery. Our finding revealed that women who follow ANC less than four times were about two times more to give preterm delivery as compared to women who attended ANC four times or more. This is consistent with the study done in Tanzania [20], Debremarkos Town Health Institution [17], western China [21], Gaza strip [22] and south-western Nigeria [23]. This could be due to the fact that women with regular ANC follow up may have more chance to early detection of severe diseases or obstetrics complications and appropriate case management. The regional government should enhance the awareness of women in ANC follow-up by increasing community ownership through women development army.

Our study revealed that mothers who had pregnancy induced hypertension had three times increased the risk of having a preterm birth than those mothers without pregnancy-induced hypertension. This finding is in line with studies conducted in Gondar [16], Debremarkos [17], southern India [19], western China [21] and south-western Nigeria [23]. This could be due to hypertension decreases the uteroplacental blood floor which leads to intrauterine growth restriction that causes preterm delivery. The other reason might be due to complications of pregnancy-induced hypertension can cause vascular damage to the placenta, which induces the oxytocin receptors, results in preterm labor and delivery.

We found that mothers with multiple pregnancies had more than twofolds increasing the likelihood of having preterm delivery as compared to mothers with singleton delivery. This is consistent with the study done in Tanzania [20], south-western Nigeria [23]. Palestine [24], southern India [19], Gaza strip [22]. This is probably due to multiple pregnancies could over distend the uterus which stimulates early labor leading to preterm delivery.

In our finding the likelihood of preterm delivery among birth defect neonate were three times more significant than those who had no any birth defect. This could be due to the interaction of genetic and environmental risk factors contribute to preterm delivery. Identification of specific modifiable risk factors should be an important research and public health priority.

In this study, we find that fetal distress was about four times more significantly associated with preterm delivery. This finding is consistent with the study done in Jakarta [25]. Fetal distress infant could medically indicated to premature births as emergency births, where the decision to deliver must be made very quickly. Pregnancy-induced hypertension may decrease blood flow from mother’s placenta which leads to hypoxemia in the fetus. This condition can lead to fetal distress.

### Conclusion

The commonest obstetrical risk factor for preterm birth was hypertensive disorders of pregnancy and multiple pregnancies. Regarding to neonates, fetal distress and birth defect were the significant variables with preterm delivery. Early detection and treatment of diseases or disorders among pregnant women as well as the improving health care quality delivered to pregnant women may reduce risk factors for preterm delivery.

## Limitation

Our study is limited to hospital-based quantitative study. It would be better if qualitative approach study was triangulated to investigate further factors on preterm birth.

## Abbreviations

AOR: adjusted odd ratio
COR: crudes odd ratio
SPSS: Statistics Package for Social Science
ANC: antenatal care
PTB: preterm birth
